# Spontaneous Coronary Artery Dissection and COVID-19: A Review of the Literature

**DOI:** 10.3390/life14030315

**Published:** 2024-02-28

**Authors:** Grigorios Tsigkas, Maria Bozika, Kassiani-Maria Nastouli, Anastasios Apostolos, Michaela Routoula, Athanasia-Maria Georga, Anastasia Latta, Angeliki Papageorgiou, Michail I. Papafaklis, Georgios Leventopoulos, Grigoris V. Karamasis, Periklis Davlouros

**Affiliations:** 1 Department of Cardiology, University Hospital of Patras, 265 04 Patras, Greece; mariabozika1604@gmail.com (M.B.); kassienmarie@gmail.com (K.-M.N.); michaeiaroutouia@gmail.com (M.R.); marisiag15@gmail.com (A.-M.G.); anastacialatta@gmail.com (A.L.); aggelikip.1502@gmail.com (A.P.); m.papafaklis@yahoo.com (M.I.P.); levent2669@gmail.com (G.L.); pdav@upatras.gr (P.D.); 2 First Department of Cardiology, Hippocration General Hospital, National and Kapodistrian University of Athens, 157 72 Athens, Greece; anastasisapostolos@gmail.com; 3 Second Cardiology Department, Attikon University Hospital, Medical School, National and Kapodistrian University of Athens, 124 62 Athens, Greece; grigoris.karamasis@gmail.com

**Keywords:** spontaneous coronary artery dissection (SCAD), coronavirus disease 2019 (COVID-19), cardiovascular manifestations, acute myocardial infraction (AMI)

## Abstract

SARS-CoV-2 is responsible for the global coronavirus disease 2019 (COVID-19) pandemic. While the cardiovascular effects of COVID-19 have been thoroughly described, there are limited published studies in the literature establishing a connection between spontaneous coronary artery dissection (SCAD) and COVID-19. Cardiovascular manifestations include, among others, myocarditis, acute myocardial infraction, and thrombosis. In general, SCAD is an uncommon and underdiagnosed cause of acute myocardial infarction (AMI), particularly in younger women and in patients with underlying fibromuscular dysplasia (FMD). Many patients with SCAD often report significant emotional stress, especially in relation with job loss, during the week preceding their cardiac event. Moreover, the COVID-19 pandemic has led to societal stress and increased unemployment, factors that have been associated with cardiovascular morbidity. SCAD emerges as a rare manifestation of coronary artery disease, which a few recent case reports link to COVID-19. The aim of this article is to summarize the relevant data on the pathophysiology of COVID-19 and SCAD along with a review of the reported cases on acute coronary syndrome (ACS) following SARS-CoV2 infection and, thus, to provide insights about the relationship between COVID-19 and SCAD.

## 1. Introduction

Since December 2019, when the first patient was diagnosed with SARS-CoV-2, more than 700 million patients have been infected with COVID-19, and about 7 million patients have died due to SARS-CoV-2 [1].

COVID-19 extends its impact beyond the respiratory system, affecting various aspects of the cardiovascular system [2]. SARS-CoV-2 mediates vascular damage through the angiotensin-converting enzyme 2 (ACE-2) receptor. This receptor facilitates viral entry into host cells, causing endothelial dysfunction, hypercoagulability, and inflammation [3]. Cardiovascular manifestations include acute coronary syndrome, arrythmias, cardiogenic shock, and cardiomyopathy [4]. The mechanisms involved are complex, involving an interplay between systematic inflammation, direct viral effects on myocardial cells, and vascular infection [5]. Emerging evidence suggests that direct viral involvement in coronary vessels induces plaque formation, thereby increasing the risk of myocardial infarction. Macrophages and foam cells within plaques are susceptible to SARS-CoV-2 infection, leading to inflammation and plaque destabilization [6].

The prevalence of SCAD reaches up to 4% of patients with an acute coronary syndrome (ACS) [7]. The presentation of SCAD is likely associated with a number of factors including age, sex, underlying pathologies, genetics, hormonal alterations, and physical, mental, and environmental triggers. SCAD is more often evoked in females, with from 87% to 95% of SCAD cases occurring with one or no cardiovascular risk factors, with mean ages being from 44 to 53 years old, while a prevalence of 35% has been reported among women with an ACS younger than 50 years old. The predisposing factors for the occurrence of SCAD include pregnancy, usually postpartum, as well as the usage of contraceptive pills [7]. Hypertension and dyslipidemia are also present in approximately 30–40% of SCAD patients. Arteriopathies, such as fibromuscular dysplasia, connective tissue disorders (Marfan’s syndrome, Ehlers–Dalton, etc.), and emotional or physical stressors are reported to be a substrate for the occurrence of SCAD [8].

The aim of this article is to summarize relevant data on the pathophysiology of COVID-19 and SCAD along with a review of the reported cases on ACS following SARS-CoV-2 infection and, thus, to provide insights about the relationship between COVID-19 and SCAD [4].

## 2. Materials and Methods

A review was conducted utilizing the PubMed and Google Scholar databases. We used the following key words: SCAD, COVID-19, acute myocardial infraction, pathophysiology, medical therapy, and case reports. The search was conducted from 1 December 2022 to 11 December 2023 and was limited to articles published in English.

## 3. Results

### 3.1. The Pathophysiologic Pathways of SARS-CoV-2 in Vascular Damage: The Role of ACE-2

SARS-CoV-2 belongs to the family of *Orthocoronavirinae*, specifically in the subgenus of betacoronaviruses. It is a positive-sense single-stranded RNA (+ssRNA) with a linear segment. The viral RNA encodes a set of proteins, structural and non-structural, with four major proteins: the spike (S) protein, which is a crucial particle for its pathogenesis, and the nucleocapsid (N), the membrane (M), and the envelope (E) ones [9]. The spike protein consists of two domains, S1 and S2. The S1 domain is responsible for the binding of the virus with the membrane of the host cells by binding to the receptor ACE-2, and the S2 subunit is responsible for membrane fusion. Additionally, the type 2 transmembrane serin protease (TMPRSS2), which is a membrane protein expressed in the endothelial cells of the respiratory and digestive tracts, promotes the entrance of the virus in the cells by cleaving ACE-2 and activating the spike protein. Both ACE-2 and TMPRSS2 are found in the alveolar epithelial type II cells, thus explaining the virus tropism for the lungs [10,11].

ACE-2 has been found to be a crucial component of the pathogenesis of COVID-19. Liet al. discovered that ACE-2 is a functional receptor for SARS-CoV-2 [12]. ACE-2 is a part of the renin angiotensin aldosterone system (RAAS) and is a multifunctional protein that mainly converts angiotensin II to Ang(1–7) and angiotensin I to Ans(1–9) [13]. ACE-2 is located in the vascular endothelium of the lungs and has the opposite functions to ACE, promoting vasodilation and decreasing hypertension, thus having a cardio-protective role [14]. ACE-2 is also highly expressed in the mouth on the tongue as well as in the nasal mucosa, and this explains the entry of the virus inside the body. The virus’ primary targets inside the lungs are the squamous epithelial cells in the alveoli due to their high expression of ACE-2 and TMPRSS2. Additionally, a newly identified protein, neuropilin (NRP1), can profoundly increase the infectivity of the virus when it is co-expressed with ACE-2 and TMPRSS2. When the virus enters the squamous cells, it replicates, and the cells undergo apoptotic cell death. Pro-inflammatory cytokines increase, leading to the recruitment of leukocytes, reinforcing the local inflammation. This stage of the inflammation induces a cytokine storm with increased levels of pro-inflammatory cytokines such as interleukin-6 (IL-6), IL-1β, and IL-18, increased levels of chemokines and granulocyte-macrophage colony-stimulating-factor (GM-CSF), and also elevated levels of macrophages and neutrophils [15,16].

The acute inflammatory effect elicited by the virus induces a prothrombotic state with hypercoagulability, endothelial dysfunction, and platelet activation. SARS-CoV-2 affects the endothelial cells directly by fusing to the ACE-2 receptor, leading to cell damage and apoptosis and reducing their antithrombotic activity. The downregulation of ACE-2 receptors activates the kallikrein–bradykinin pathway, and, at the same time, the release of inflammatory cytokines and vasoactive molecules result in a loosening of the intraepithelial junction, leading to endothelial gaps. Additionally, there is a proliferation of hyaluronic acid due to the activity of IL-1β and TNF, thereby leading to fluid retention in the extracellular matrix. Hence, the vascular integrity is disrupted, and the endothelium is prone to inflammation and immunothrombosis, which is observed in the majority of patients suffering from COVID-19 [17].

All the data imply that the effect of the disease on the human body is not only restricted to the lungs and that ARDS is not the only critical clinical manifestation. Multiple extrapulmonary manifestations can be presented, including cardiovascular manifestations such as acute coronary syndrome (ACS), arrhythmias, cardiogenic shock, and cardiomyopathy, especially in patients with pre-existing heart conditions [18].

The underlying etiology between COVID-19 and myocardial manifestations is not yet well defined. Multiple factors lead to the appearance of cardiac manifestations. ACE-2 is highly expressed in myocardial cells in a comparable level to that in lung tissue. The myocardial injury caused by the virus may be connected with systemic inflammation. ACS may occur either from plaque rupture, microthrombi that result from systemic inflammation due to the coagulopathy that is observed, from a coronary spasm, or from the cytokine storm that affects the heart indirectly, leading to an imbalance in the supply and demand of oxygen in the myocardium. The direct effect of the virus in the vessels can also affect the risk for atherosclerotic plaque formation [19].

Eberhardt et al. recently showed that COVID-19 can directly infect the coronary vessels and that it can induce a pro-inflammatory state, leading to the observed cardiovascular manifestations [6]. SARS-CoV-2 was shown to be accumulated in macrophages and especially foam cells, which are critical for the formation of atherosclerotic plaques. The investigation showed that the viral RNA levels were cleared faster in the macrophages than in the foam cells and that the virus replicates easier in them due to the reduced IFN type I response. It was noted that the macrophages were more prone to infection from the virus in the early stages of atherosclerosis, where the numerous dysfunctional lipids are accumulated by macrophages, and not in progressed stages of atherosclerosis, when the inflammatory environment is harmful for the correct function of the cells [6].

Direct infection of the atherosclerotic plaque by SARS-CoV-2 produces an enhanced inflammatory response with the release of cytokines such as IL-6 and IL-1β, which are key to the formation of a plaque. Plaque rupture can be caused due to the inflammation, leading to myocardial infarction and ACS. The coronary arteries are more prone to viral infection due to the high expression of ACE-2. The existence of NRP1+ macrophages enhance the inflammation of the coronary vessels, increasing cardiovascular risk and myocardial infarction. Overall, the hyperinflammatory environment produced by the virus in the macrophages and foam cells of the plaque may be a connection between acute cardiovascular manifestations and vessel infection [6].

### 3.2. Pathophysiology of SCAD

SCAD, a non-traumatic, non-iatrogenic spontaneous rupture of the coronary wall, is a cause of ACS and sudden death. In many cases, SCAD is an underdiagnosed and underrecognized condition that can lead to myocardial infarction (MI). The coronary arteries are composed of three layers: the tunica intima, tunica media, and tunica adventitia. The tunica intima is the lining inside the vessels, comprising endothelial cells; the internal elastic lamina separates the intima one from the media one, which comprises smooth muscle cells; and the external elastic lamina separates the media from the tunica adventitia, which comprises connective tissue, nerves, and blood vessels (vasa vasorum). MI occurs when the separation of the intima generates a false lumen and the expansion of intramural hematoma (IMH) causes occlusion of the true lumen [20].

There are two main hypotheses for the underlying pathophysiology of SCAD. The first is referred to as “inside-out”, and it suggests that blood enters the sub-intimal space because of an endothelial tear or “flap”. The second hypothesis is called “outside-in”, suggesting that an IMH arises de novo in the media. This knowledge is based on the following observations: (1) most of the times SCAD cases do not appear to have any communication between true and false lumens, (2) IMH can be seen before the development of intimal dissection, and (3) intravascular imaging with optical coherence tomography (OCT) indicates that the compression of the false lumen is high and that the blockage(s) can be the result of the rupture of the false lumen into the true lumen [21].

The role of inflammation and the abnormalities of connective tissue may also be implicated in the pathophysiology of SCAD. Histopathological analyses of SCAD in autopsy reports show that the outer connective tissue layer of the affected coronary artery includes cells of the inflammatory infiltrate; however, the relationship with SCAD is not clear. The latest study comparing autopsy-diagnosed SCAD cases with SCAD survivors showed that there is greater proximal left-coronary involvement in the former group, and this supports the fact that the severity of symptoms and the risk of MI leading to sudden cardiac death depends on the location of the arteries affected by SCAD and the severity of the blockage(s) [22].

### 3.3. Association of COVID-19 with SCAD: Possible Pathophysiologic Mechanisms

**Figure 1:**
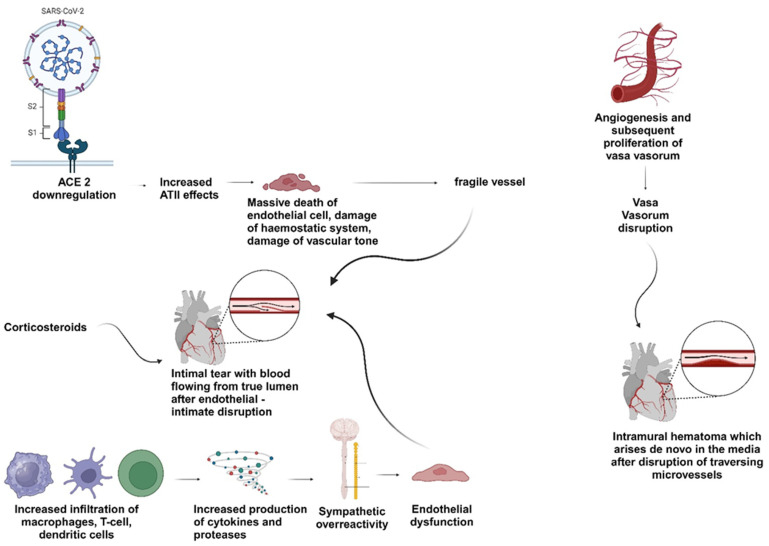
Diagram illustrates the underlying pathogenesis of SCAD related to COVID-19. Created with Biorender.com (accessed on 18 February 2024).

There are several theories about the pathophysiologic mechanisms of SCAD among COVID-19 patients, indicating that it is potentially a multifactorial complex process (Figure 1). It has been suggested that intense inflammation in response to the infection and endothelial dysfunction can cause sympathetic over-reactivity that can lead to intimal dissection. In patients with ACS, there is an increased infiltration of macrophages, T-cells, and dendritic cells in the adventitia, intima, and in the media layers of coronary vessels [23]. SARS-CoV-2 viral infection may cause infiltration and activation of T-cells in the adventitia and periadventitial fat, causing the production of cytokines and proteases. The large number of cytokines and proteases in the arterial wall increases the likelihood of plaque rupture or erosion, which can lead to dissection (inside-out mechanism of SCAD) [21]. In the context of COVID-19, another pathophysiological mechanism has been suggested, which includes the stimulation of angiogenesis and the subsequent proliferation of vasa vasorum. An intramural hematoma can occur because the newly formed and fragile vasa vasorum tend to rupture (outside-in mechanism of SCAD) [24]. In addition, the vasa vasorum can act as a conduit through which inflammatory cells will enter the medial and adventitial layers of the vessel, ultimately leading to the rupture of the vasa vasorum [25]. SARS-CoV-2 can itself prompt inflammation in the vessel wall, as the virus can directly enter the coronary arteries using the ACE receptors. Therefore, the endothelial cells will experience massive death and the hemostatic system and the vascular tone will be damaged, which finally makes the vessel wall fragile and can result in dissection. Lastly, the treatment of some patients with high doses of corticosteroids, which are commonly used in COVID-19 patients, may induce spontaneous rupture of the weakened arterial wall [26].

### 3.4. Special Considerations Regarding SCAD following COVID-19 Infection and Heart Involvement

Although SCAD is more common in females, six out of twelve cases of SCAD presentation after COVID-19 involved males (Table 1); this observation could be explained by the higher morbidity associated with COVID-19 in males compared to females [27]. The most common symptom was chest pain, but four patients [28,29,30,31] had different symptoms and no chest pain. Most patients also had symptoms related to SARS-CoV-2 infection such as fever, dyspnea, and cough, which are not characteristic of acute coronary events. Of note is the fact that most patients with SCAD (n = 9) experienced mild symptoms related to COVID-19 [26,29,30,32,33,34,35,36,37], five of them faced more severe manifestations [28,31,38,39,40], and only two demonstrated no symptoms [41,42].

In the majority of the cases presented, patients with SCAD were diagnosed with myocardial infarction. Specifically, eight out of twelve patients were diagnosed with non-ST-segment-elevation myocardial infarction (NSTEMI) [28,31,32,34,35,36,39,42], while the other seven patients had ST-elevation myocardial infarction (STEMI) [26,29,33,37,38,40,41]. In the majority of patients (10 out of the 12 cases), the vessel involved was the left anterior descending coronary artery [28,31,33,34,35,37,38,40,41,42]; the right coronary artery was affected in two patients [29,32], the circumflex in four patients [30,36,38,40], and the ramus intermedius in one patient [31]. Among these, two patients had multivessel dissection [26,38]. According to the data presented, nine of the sixteen cases of SCAD occurred in the immediate acute phase of the SARS-CoV-2 viral infection [29,32,34,35,37,39,40,41,42], while the other seven cases occurred in the post-acute phase (>14 days after the infection) [26,28,30,31,33,36,38]. A total of four patients underwent percutaneous coronary intervention (PCI) during their SCAD hospitalization [26,29,34,41], twelve patients were treated medically [28,30,31,32,33,35,36,37,38,39,40,42], and none of the patients underwent coronary artery bypass grafting (CABG).

In addition to the documented instances of spontaneous coronary artery dissection (SCAD) with AMI following SARS-CoV-2 infection, there were also two cases of SCAD occurring after COVID-19, as reported by Mohammed et al. [27]. The first patient, a 58-year-old woman, was admitted two days after vaccination with severe chest pain, high troponin levels, and positive inflammatory markers. The patient had a history of arterial hypertension and dyslipidemia. She was diagnosed with NSTEMI; thus, she underwent coronary angiography. The intravascular ultrasound showed the dissection of the LAD. She was treated conservatively with statins and a single antiplatelet agent, due to the patency of the vessel. The second patient, a 48-year-old woman, was admitted 3 days after SARS-CoV-2 vaccination. During the coronary angiography, the distal circumflex artery was diffusely narrowed. An optical coherence tomographic analysis was performed and confirmed the SCAD diagnosis. The patient was treated conservatively due to the patency of the vessel.

These cases suggest a potential association between SARS-CoV-2 vaccination and SCAD, proposing that the vaccine may trigger an inflammatory response, leading to the production of cytokines and complement factors. In genetically predisposed individuals, this immune response could trigger systemic inflammation similar to COVID-19 infection, potentially leading to SCAD. It is possible that the mechanism that leads to SCAD after vaccination is immune-mediated by cytokines that disrupt the tunica intima, leading to bleeding [27].

Acknowledging the rarity of SCAD occurrences post-vaccination, the study refrains from making definite causal claims. The findings of the study emphasize the need for further research to establish the final relationship between SARS-CoV-2 vaccination and SCAD [27].

### 3.5. Diagnosis of SCAD—Clinical Presentation

The true prevalence of SCAD cannot be determined with absolute precision due to underdiagnosis. The clinical presentation of SCAD is almost identical with atherosclerotic ACS, and this is the reason why the diagnosis of SCAD requires a strong index of suspicion. The majority of patients with SCAD present in the ER with chest pain, and they tend to have elevated biomarkers and ECG changes compatible with STEMI or NSTEMI. The gold standard in order to confirm the presence of SCAD is coronary angiography (CAG), but in some cases it may be difficult to differentiate a potential case of SCAD from other causes of coronary artery stenosis. Even using intracoronary angiography imaging, the type III subtype of SCAD could be confused with a focal ruptured atheromatic plaque. The use of new intracoronary methods, such as OCT and intravascular ultrasound, has led to a maximized diagnostic accuracy and to the ability to delineate hematoma and intimal tear or even exclude an atherosclerotic plaque rupture [43].

### 3.6. Management of Patients with COVID-19 and SCAD

#### Conservative Pharmacotherapy versus Invasive Management

The target of SCAD therapy is to reduce patients’ symptoms and to improve both short- and long-term prognosis and intercept recurrence. The management of a STEMI-SCAD patient can be complex, and it can vary based on the patient’s clinical presentation. In general, the first-line therapy is the conservative therapy, while revascularization is recommended for patients who have high-risk features such as ongoing ischemia, ventricular arrhythmias, left main coronary artery dissection, or hemodynamic instability. PCI is the dominant method used in ACS, but compared with the atherosclerotic AMI, the PCI in SCAD patients seems to have higher rates of complications. First of all, iatrogenic dissection can easily occur during the procedure due to the fragility of the arterial wall. If an intimal rupture is present, then it can be difficult to advance the coronary wire into the true lumen. In addition, the propagation of hematoma due to the malapposition of a stent requires the use of multiple or long stents that can lead to an increased risk of stent thrombosis and restenosis. Furthermore, the angioplasty and stent implantation to distal small coronary segments may be challenging. When revascularization therapy is needed, PCI should be performed, with coronary artery bypass grafting (CABG) serving as a “bail-out” option if the anatomy is not compatible with PCI [44]. Tweet et al. [45] evaluated the outcomes of twenty patients who were treated with revascularization by CABG. The early mortality was significant at 5%, but there was no death at 5-year follow up. At a median follow up angiography of 3.5 years, only 5 of 16 grafts were patent. This can be explained by the competitive flow in a healed native artery, leading to graft occlusion and contributing to high rates of bypass failure. Despite these observations, CABG should be an important revascularization option for selected SCAD patients in order to reduce serious clinical complications and provide the patient an excellent short- and long-term clinical outcomes post CABG.

At the outset of the COVID-19 pandemic, in order to limit nosocomial cross-infection, the guidelines recommended that thrombolytic therapy should be the most favored therapy for patients with ST-segment-elevation myocardial infarction (STEMI) [46]. However, thrombolytic therapy in SCAD is related with the extension of dissection and may lead to coronary rupture and cardiac tamponade [47]. As the medical community gained more experience dealing with the issues raised by the COVID-19 pandemic, the guidelines changed, and PCI remained the standard of care for STEMI patients at PCI-capable hospitals [48]. Regarding the medical treatment, SCAD survey responders prefer aspirin, P2Y12 inhibitors, beta-blockers, and statins as a first-line treatment, followed by angiotensin-converting enzyme inhibitors, nitrates, calcium channel blockers, and angiotensin receptor blockers, respectively [49]. Dual antiplatelet therapy (DAPT) is not favorable in SCAD patients because intramural bleeding appears to be the initiating pathophysiological event and, therefore, this therapy can increase the risk of bleeding. A comparison between SAPT and DAPT proved that DAPT was related with a higher risk of MACE at 1-year follow up. DAPT is recommended in cases of patients who have undergone PCI and should be continued for 1 year followed by SAPT [50].

The hypercoagulable state in COVID-19 remains a major concern among physicians treating patients with COVID-19 [51]. Two patients received dual antiplatelet therapy (DAPT) after conservative treatment [37,38], while only aspirin (ASA) was prescribed for two others [26,30]. Two patients received DAPT after angioplasty [29,34], and one, following plain old balloon angioplasty (POBA), was discharged with ASA alone [41].

Regarding anticoagulation therapy in SCAD, preventing thrombus formation may be beneficial, but it poses a risk of intramural bleeding and extending the dissection. Thus, the therapeutic approaches vary widely among the recorded cases. In Papanikolaou et al.’s case report, a patient received anticoagulation with DAPT, seemingly linked to COVID-19 infection [39]. Papageorgiou et al. opted for a preventive dose of rivaroxaban with single antiplatelet therapy to prevent thromboembolism during the acute phase [40]. Kireev et al. administered DAPT and intravenous unfractionated heparin despite the presence of SCAD [26]. Pettinato et al. [31] and Bashir et al. [35] used ASA, clopidogrel with warfarin, and clopidogrel with warfarin, respectively, for an apical thrombus, with no reported adverse events. The optimal post-SCAD anticoagulation treatment remains unclear, emphasizing the need for future studies.

## 4. Discussion

The association between COVID-19 and SCAD is not well established, and while there is a theoretical possibility that inflammation associated with COVID-19 could increase the risk of SCAD, it is challenging to draw definitive conclusions. The difficulty likely arises from various factors, such as the complexity of studying rare events like SCAD, the multifaceted nature of COVID-19’s impact on the body, and the need for large-scale, well-designed studies to establish a causal relationship between the two conditions.

While SCAD typically affects females more frequently, there is a notably discernible shift in gender distribution, with a higher proportion of male patients presenting with SCAD related to COVID-19. This shift may reflect gender-specific morbidity patterns seen in COVID-19, possibly attributed to the higher reported incidence of COVID-19 among males [52].

Diagnostic intricacies associated with SCAD are highlighted, emphasizing the limitations of traditional angiography and the utility of adjunctive imaging techniques, such as intravascular ultrasound and optical coherence tomography in enhancing diagnostic accuracy. The medical management of patients with COVID-19 and SCAD poses dilemmas. The heightened risks associated with revascularization procedures in SCAD patients underscore the need for individualized treatment strategies based on clinical presentation and risk assessment.

Furthermore, the American Heart Association (AHA) emphasizes the importance of addressing mental health in patients recovering from SCAD due to high rates of anxiety and depression [52]. The COVID-19 pandemic has worsened societal stressors, leading to increased rates of major depressive disorder and generalized anxiety disorder. Pandemic-related stressors, including unemployment, may contribute to medical pathologies with stress-induced etiologies, such as Takotsubo cardiomyopathy and SCAD. Emotional stressors should be managed properly and may be crucial in the treatment of SCAD related to COVID-19 [52].

## 5. Conclusions

COVID-19 infection is a clinical syndrome that affects multiple organs besides the lungs. The growing understanding of SARS-CoV-2 infection’s pathogenesis and how it may contribute to SCAD may help to improve diagnosis and risk stratification. Currently, there are no guidelines on the management of COVID-19 patients with SCAD. As the number of patients with COVID-19 increases, further research is needed to clarify the best treatment options in the subgroup of patients with SCAD.

## Figures and Tables

**Table 1:** Review of case reports on spontaneous coronary artery dissection (SCAD) associated with COVID-19.

Number	Author, Published Date	Country	Sex	Age	Symptoms	Findings from Lab and Imaging Tests	Past Medical History/ Predisposing Factors	Timing According to COVID-19 Infection	COVID-19 Severity	Complications Associated with COVID-19 Infection	Diagnosis	Arteries	Treatment	Outcome
1	Courand et al., 8 April 2020 [32]	France	Male	55 years old	Cough, febrile dyspnea, chest pain	Abnormal ECG, elevated Hs-TnI, echo: LVEF = 60%, mild mitral regurgitation	Peripheral artery disease/ unreported	48 h after a positive COVID-19 test	Moderate, crazy pavy pattern in the lung	None	Non-STEMI	RCA → mild dissection PDA → chronic total occlusion with epicardial collaterals	Conservative aspirin, statins, B-blockers	Survived
2	Kumar et al., 7 May 2020 [37]	USA	Female	48 years old	Chest pain	First hospital admission: elevated troponin, normal ECG 3 days post discharge: elevated troponin, abnormal ECG	Migraines, hyperlipidemia/ unreported	COVID-19 test (+) 8 h after SCAD had been diagnosed	Mild	Polymorphic ventricular tachycardia	STEMI	LAD	Conservative dual antiplatelet, beta blocker, amiodarone	Survived
3	Gasso et al., 7 May 2020 [38]	Spain	Male	39 years old	Fever, cough, myalgia chest pain, dyspnea	Elevated ferritin, CRP, LDH. 13 days after admission → elevated troponin levels, abnormal ECG, echo → LVEF = 50%		18 days	Severe intubation because of respiratory failure	None	STEMI	LAD, LCx	Conservative dual antiplatelet treatment	Survived
4	Albiero et al., 12 May 2020 [34]	Italy	Male	70 years old	Severe chest pain	Increased D-dimers abnormal ECG, echo: left-ventricular wall motion abnormalities	Hypertension, type 2 diabetes, prior PCI to LCx/smoking, hypertension	1 day after positive test	Mild	None	Non-STEMI, ACS	LAD → A-SCAD, LCx-OM → in stent restenosis RCA → moderate stenosis	PCI → LAD aspirin, clopidogrel, pantoprazole, atorvastatin, bisoprolol, metformin	Survived
5	Kireev et al., 27 November 2020 [26]	Russia	Male	35 years old	Pressure chest pain, fever, dry cough, nasal congestion, chest congestion	CRP elevation, abnormal ECG	Obese, smoker/ autoimmune diseases were ruled out	Approximately 18 days	Mild	None	STEMI	PCI → RI, RCA occluded distal, extended lesions, control angiography → RCA and RI unchanged, stenosis in an RI branch	PCI → RI conservative	Survived
6	Cannata et al., 18 December 2020 [33]	Great Britain	Female	45 years old	Chest pain, anosmia, hypogeusia	Abnormal ECG	None/ unreported	8 weeks	Mild	None	STEMI	LAD	Conservative dual antiplatelet therapy, B-blockers, ACE-I	Survived
7	Aparisi et al., 21 December 2020 [28]	Spain	Male	40 years old	Fever, cough	Elevated troponin-T, D-dimers, CRP, ferritin, lymphopenia	None/ unreported	8 weeks	Severe lung infiltration	Cardiogenic shock, severe respiratory distress syndrome, cardiac thrombus	Non-STEMI	LAD	Conservative therapy aspirin, guideline-directed medical therapy for HF; the follow-up CA confirmed the complete resolution	Survived
8	Papanikolaou et al., 23 December 2020 [39]	Saudi Arabia	Female	51 years old	Fever, cough, respiratory distress, chest pain	Abnormal ECG, positive cardiac enzymes	Hypertension/ unreported	3 days	Mild	None	Non-STEMI	LAD	Conservative therapy, dual antiplatelet, anticoagulation, statin	Survived
9	Emren et al., 1 June 2021 [29]	Turkey	Male	50 years old	Cough, fever, chest pain (later)	Abnormal ECG	None/ unreported	7 days	Mild	None	STEMI	RCA	PCI, dual antiplatelet, atorvastatin, metoprolol	Survived
10	Ahmad et al., 11 October 2021 [30]		Female	43 years old	Syncope	Elevated troponin	Atrial fibrillation	12-weeks-prior positive PCR test	Severe	Intubation	Cardiogenic shock	LCX	Conservative	Survived
11	Pettinato et al., 1 July 2022 [31]	USA	Female	43 years old	Initial hospital admission: fever, weakness, dysphagia, vomiting, diarrhea, maculopapular rash. Second hospital admission (2 months later): chest tightness, nausea	First hospital admission: acute renal failure, lactic acidosis, hypotension, leukocytosis, elevated CRP, ferritin, D-dimers, left-side colitis, oral candidiasis, antibody-negative hypothyroidism. Second hospital admission: abnormal ECG, elevated troponin, D-dimer, LDL	None/ unreported	PCR-positive test 3 months prior to dissection	Mild	Multisystem inflammatory syndrome (MIS-A) 1 month after SARS-CoV-2 infection	Non-STEMI	LAD	Conservative antiplatelet, clopidogrel, carvedilol, metoprolol, spironolactone, lisinopril, atorvastatin, warfarin	Survived
12	Ansari et al., 3 October 2022 [36]		Female	58 years old	Chest pain	Abnormal ECG, leucocytosis, high CRP, increased hs-cTnI	Hyperlipidemia/ unreported	Approximately 2 months after the active phase of COVID-19	Fever, gastrointestinal symptoms, isolate severe thrombocytopenia	None	Troponin-positive chest pain with pericardial components	LCx	Conservative antiplatelet, clopidogrel, metoprolol, lisinopril, statin	Survived
13.	Lewars et al., 19 October 2022 [42]		Female	51 years old	Chest pain, dyspnea	Elevated troponin, First ECG: consistent with acute ischemic changes, Second ECG: Abnormal ECG	Anxiety, postpartum cardiomyopathy 15 years prior with recovered ejection fraction/ unreported	24 h after result	Asymptomatic	None	Troponin-positive chest pain	LAD	Conservative	Survived
14.	Bashir et al., 15 April 2023 [35]		Female	36 years old	Severe chest pain.	Elevated troponin, hyperacute T on ECG RBBB	Morbid obesity	A few hours before the PCR test returned positive	Mild, fever	None	NSTEMI	LAD	Conservative	Survived
15.	Shah et al., 24 June 2023 [41]		Female	67 years old	Chest pain, shortness of breath, nausea	First ECG: abnormal ECG Second ECG: abnormal ECG, elevated troponin	None/FHCAD	1 day before a positive PCR test	Asymptomatic	None	STEMI	LAD	POBA	
16.	Papageorgiou et al., 16 January 2024 [40]	Greece	Male	51 years old	Chest pain	Abnormal ECG	Hypertension	7 days after a positive PCR test	Fever, severe	Severe respiratory distress	STEMI	LCX	Conservative	Survived

ECG: electrocardiogram; HS-TnI: high-sensitivity troponin I; LVEF: left-ventricular ejection fraction; non-STEMI: non-ST-elevation myocardial infarction; STEMI: ST-elevation myocardial infarction; LAD: left anterior descending artery; LCx: left circumflex artery; CRP: C-reactive protein; LDH: lactate dehydrogenase; OM: otitis media; RI: ramus intermedius; PCI: percutaneous coronary intervention; RCA: right coronary artery; POBA: percutaneous old balloon angioplasty; RBBB: right bundle branch block; PCR: polymerase chain reaction.

## Data Availability

Not applicable.
